# Geniposide alleviates cholesterol-induced endoplasmic reticulum stress and apoptosis in osteoblasts by mediating the GLP-1R/ABCA1 pathway

**DOI:** 10.1186/s13018-024-04665-4

**Published:** 2024-03-11

**Authors:** Mingliang Zhong, Zhenyu Wu, Zhixi Chen, Longhuo Wu, Jianguo Zhou

**Affiliations:** 1 College of Rehabilitation, Gannan Medical University, Ganzhou, 341000 China; 2 First Affiliated Hospital of Gannan Medical University, Ganzhou, 341000 China; 3 College of Pharmacy, Gannan Medical University, Ganzhou, 341000 China; 4 Department of Joint Surgery, Ganzhou People’s Hospital, Ganzhou, 341000 China

**Keywords:** Osteoporosis, Endoplasmic reticulum stress, Cholesterol, Geniposide, GLP-1R, ABCA1

## Abstract

**Background:**

Cholesterol (CHO) is an essential component of the body. However, high CHO levels in the body can damage bone mass and promote osteoporosis. CHO accumulation can cause osteoblast apoptosis, which has a negative effect on bone formation. The pathogenesis of osteoporosis is a complicate process that includes oxidative stress, endoplasmic reticulum (ER) stress, and inflammation. Geniposide (GEN) is a natural compound with anti-osteoporotic effect. However, the roles of GEN in osteopathogenesis are still unclear. Our previous studies demonstrated that GEN could reduce the accumulation of CHO in osteoblasts and the activation of ER stress in osteoblasts. However, the molecular mechanism of GEN in inhibiting CHO-induced apoptosis in osteoblasts needs to be further investigated.

**Methods:**

MC3T3-E1 cells were treated with osteogenic induction medium (OIM). Ethanol-solubilized cholesterol (100 µM) was used as a stimulator, and 10 µM and 25 µM geniposide was added for treatment. The alterations of protein expression were detected by western blot, and the cell apoptosis was analyzed by a flow cytometer.

**Results:**

CHO promoted osteoblast apoptosis by activating ER stress in osteoblasts, while GEN alleviated the activation of ER stress and reduced osteoblast apoptosis by activating the GLP-1R/ABCA1 pathway. Inhibition of ABCA1 or GLP-1R could eliminate the protective activity of GEN against CHO-induced ER stress and osteoblast apoptosis.

**Conclusion:**

GEN alleviated CHO-induced ER stress and apoptosis in osteoblasts by mediating the GLP-1R/ABCA1 pathway.

## Introduction

Osteoporosis, a common skeletal disorder, is characterized by loss of bone mass, destruction of bone microarchitecture, and high risk of fracture [1]. Osteoporosis is one of the chronic and aging diseases, and it becomes a major issue as the world’s population ages. Whether as a result of some reasons or an imbalance in estrogen metabolism, the trend is upward, and in Brazil, for instance, osteoporosis prevalence in postmenopausal women ranges from 15 to 33% [2]. In addition, studies have shown that more than 100,000 people in the European Union in 2010 suffer from osteoporosis and there is a need to explore the pathogenesis of osteoporosis and develop more drugs to treat it [3]. It is well known that osteoporosis results directly from bone loss. Under normal conditions, skeletal homeostasis is maintained in balance. Pathologically, skeletal homeostasis is disrupted and bone loss initiates. Bone formation is decreased, and bone resorption is increased.

Dyslipidemia has been related to osteoporosis and fracture risk. One study reports that dyslipidemia can induce oxidative stress, increase systemic inflammation, decrease bone formation, and increase osteoclast activity [4]. Cholesterol (CHO) is a form of lipid, and the role of hypercholesterolemia in osteoclastogenesis and bone quality reduction has been reported [5]. CHO accumulation can be harmful to bone metabolism and contribute to the development of osteoporosis [6]. It has been reported that CHO at higher than physiological levels inhibits osteogenic proliferation and differentiation and promotes bone breakdown, leading to bone loss and osteoporosis [7]. Unfortunately, the underlying mechanisms have not yet been fully clarified. Studies have shown that intracellular CHO accumulation induces endoplasmic reticulum (ER) stress. In turn, activation of ER stress further impairs cellular CHO synthesis and efflux and cell survival [8].

ER, the largest organelle in eukaryotic cells, is the site of protein folding, calcium ion storage, and CHO synthesis [9]. Under stimulations, cells activate ER stress, a cellular self-protection mechanism that attempts to restore intracellular homeostasis and avoid cellular damage by initiating the unfolded protein response (UPR). The UPR machinery included three signaling transductions, such as protein kinase RNA-like ER kinase (PERK)/eukaryotic initiation factor 2 alpha (eIF2α)/activating transcription factor 4 (ATF4) pathway, inositol-requiring protein 1α (IRE1α)/X-box binding protein-1 (XBP1), and ATF6. In homeostatic cells, 78 kDa Glucose-Regulated Protein (GRP78) is bound to UPR sensors (PERK, IRE1α, and ATF6) and keeps them inactive. Under ER stress, GRP78 is dissociated from UPR sensors, initiating UPR signaling [10]. After GRP78 dissociation, trans-autophosphorylation of PERK leads to the phosphorylation of eIF2α, which further increases ATF4 expression and decreases global protein translation. Similar to PERK, the phosphorylated dimer of IRE1α can splice XBP1 mRNA by cleaving out 26 intronic nucleotides. The generation of XBP1s can induce gene expression involved in protein folding and ER-associated protein degradation (ERAD). ATF6 become active by protease cleavage, and active ATF6 induces the gene expression involved in chaperone, XBP1, and ERAD. However, when the challenge is overwhelmed, the PERK/eIF2α/ATF4 enhances the expression of C/EBP homologous protein (CHOP), and ER stress drives cell apoptosis [11].

**Fig. 1:**
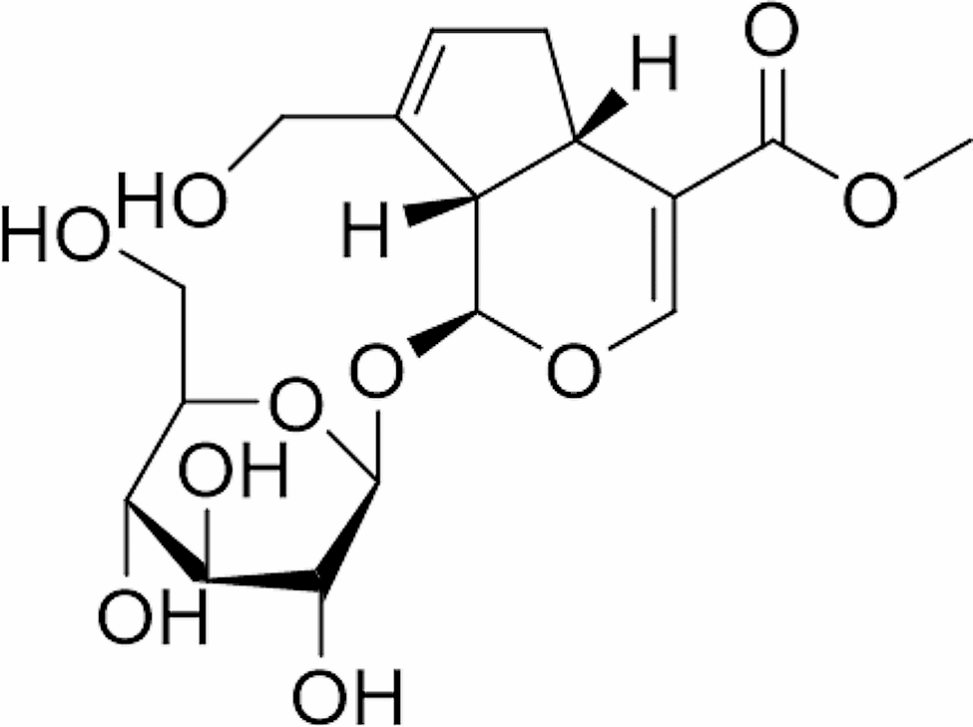
The chemical structure of GEN

It has been shown that CHO activates ER stress [12, 13], which promotes the development of osteoporosis [14]. Geniposide (GEN, Fig. 1), an active iridoid glycoside derived from *Eucommia ulmoides* Oliv [15]. and *Gardenia jasminoides* Ellis [16], has been reported to exhibit protective effects against ischemia-reperfusion injury in cardiovascular and neuronal diseases [17, 18] and therapeutic activity in chronic inflammatory diseases [19]. GEN becomes a research agent in preclinical studies related with diseases [20]. GEN has been shown to improve osteoporosis [21]. ATP-binding cassette transporter A1 (ABCA1) is a carrier for the intracellular cholesterol out of the cytoplasm [22]. GEN has been shown to improve dexamethasone-induced cholesterol accumulation and osteogenic differentiation suppression in MC3T3-E1 cells by mediating the ABCA1/GLP-1R signaling [23]. In addition, GEN exhibits protective against dexamethasone-induced ER stress [24]. However, the role of GEN in CHO accumulation-induced ER stress in osteoblasts was still unclear. In this article, we investigated whether GEN alleviated CHO accumulation-induced ER stress and reduces osteoblast apoptosis by mediating the ABCA1/GLP-1R signaling pathway.

## Materials and methods

### Cell culture

This study (GMU202204) is approved by the Ethic Committee of Gannan Medical University, according to the Declaration of Helsinki Principles. MC3T3-E1 cells were purchased from Chinese Academy of Sciences Cell Bank. Cells were cultured in α-MEM (Gibco) supplemented with 10% fetal bovine serum (FBS) (Gibco), penicillin (100 U) (Gibco), and streptomycin (100 U) (Gibco). The osteogenic induction medium (OIM) was prepared as described in our previous study [23]. Osteogenic differentiation was induced by OIM for 15 days of culture. Ethanol-solubilized CHO (100 µM) was used as a stimulant drug to stimulate osteoblasts for 72 h [23]. 10 µM and 25 µM GEN (MedChemexpress, New Jersey, USA, cat.no.HY-N0009/CS-1533) were added for 72-h treatment. The cells in the negative control groups were treated with the same volume of vehicle.

### Detection of protein expression by western blot

Cells were washed with PBS twice and collected after trypsin digestion. Cells were lysed with RIPA to collect proteins, which were used for protein assays. 30 µg of each sample was separated by 8–12% SDS-PAGE. Then, the bands were transferred onto PVDF membranes. Primary antibodies caspase-3 (1:1000 dilution; Cat.no.AF6311, Affinity), cleaved caspase-3 (1:1000 dilution; Cat.no.AF7022, Affinity), Bcl-2 (1:1000 dilution; Cat.no.AF6139, Affinity), Bax (1:1000 dilution; Cat.no.AF0120, Affinity), GRP78 (1:1000 dilution; Cat.no.AF5366, Affinity), PERK (1:1000 dilution; Cat.no.AF5304, Affinity), P-PERK (1:1000 dilution; Cat.no.DF7576, Affinity), CHOP (1:1000 dilution; Cat.no.AF6277, Affinity), IRE1α (1:1000 dilution; Cat.no.AF7651, Affinity), P-IRE1α(1:1000 dilution; Cat.no.AF7150, Affinity), and ATF6 (1:1000 dilution; Cat.no.DF6009, Affinity) were, respectively, added and co-incubated overnight at 4 °C. Next, HRP-labeled goat and rabbit secondary antibody (1:5000; Cat.no.BA1039, Boster, Wuhan, China) was added and co-incubated at room temperature for 1 h. The bands were detected by chemiluminescence imaging, and images were subsequently analyzed by ImageJ (an open-source image processing package).

### Detection of cell apoptosis

The Annexin V-FITC/PI Kit (Beyotime, Shanghai, China) is used for the detection of apoptosis by flow cytometry. After drug treatment, cells were trypsinized in 6-well plates and stained with Annexin V/FITC with PI dye for 15 min at room temperature. Samples were then detected by flow cytometry (Accuri C6 plus, Becton Dickinson, Franklin Lakes, NJ, USA).

### Statistical analysis

Data were indicated as the mean ± standard deviation (SD). Statistical analysis was conducted by the GraphPad Prism v7 software (GraphPad Software Inc., La Jolla, CA, USA). The one-way analysis of variance (ANOVA) and subsequent Bonferroni’s multiple comparisons test were analyzed. *p* < 0.05 indicated a statistical difference.

## Results

### GEN inhibits CHO-induced apoptosis of osteoblasts

**Fig. 2:**
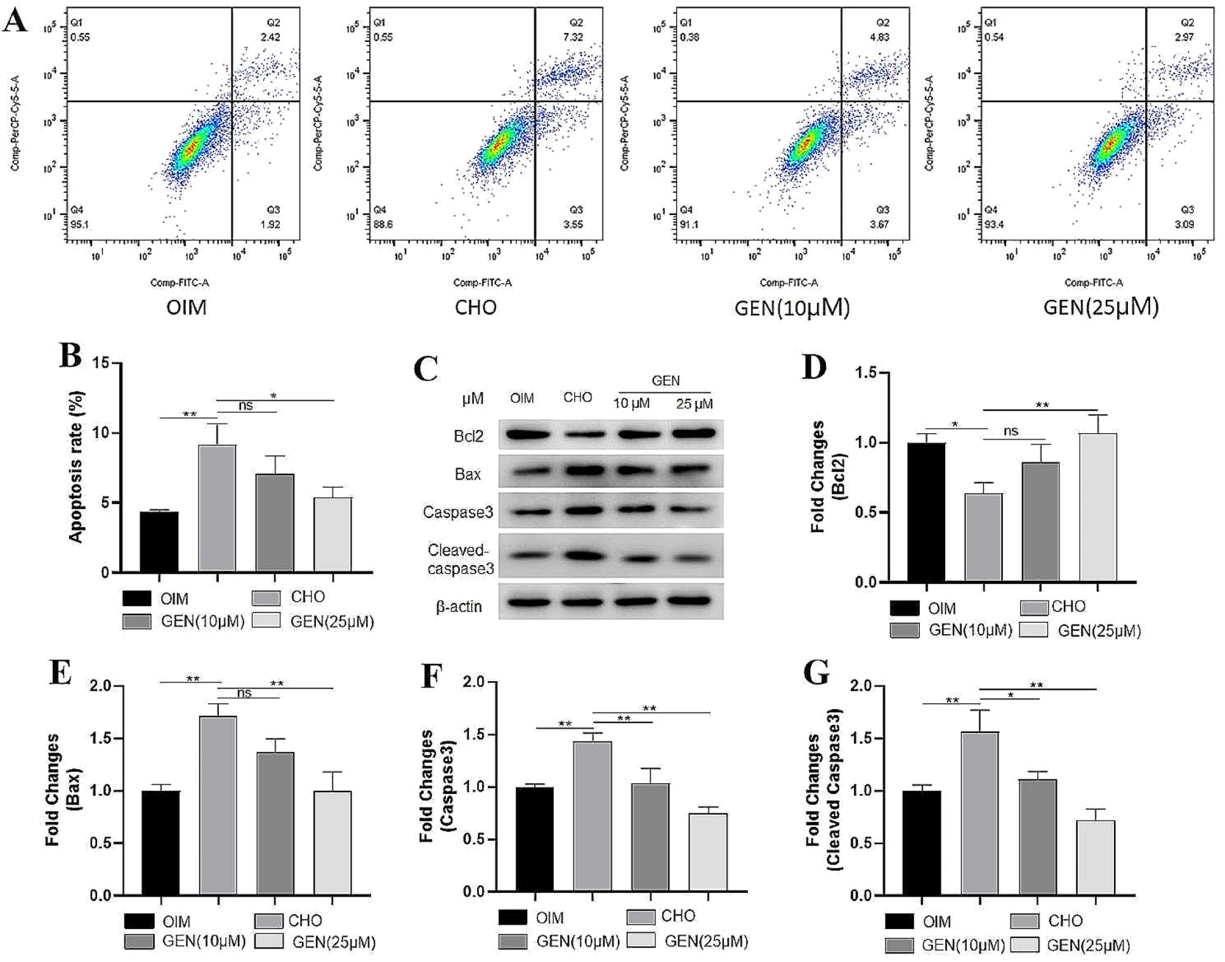
GEN inhibited CHO-induced apoptosis of osteoblasts. (**A**, **B**) Apoptosis analysis was applied by flow cytometry. The protein expressions of Bcl2 (**C**, **D**), Bax (**C**, **E**), caspase3 (**C**, **F**), and cleaved caspase3 (**C**, **G**) were analyzed by Western blot. * *p* < 0.05; ** *p* < 0.01; ns, no statistical difference. GEN (10 µM), CHO + GEN (10 µM); GEN (25 µM), CHO + GEN (15 µM)

To investigate the protective effect of GEN on CHO-treated osteoblasts, apoptosis detection and western blot assays were performed. The results showed that the apoptosis was enhanced in CHO-treated osteoblasts, compared to that in the control group. Treatment with GEN significantly reduced osteoblast apoptosis (Fig. 2A-B). In addition, the protein expression of apoptosis-related factors, such as Bax (Fig. 12C-D), caspase-3 (Fig. 2C, E), cleaved caspase-3 (Fig. 2C, F) was increased after CHO treatment, while the protein expression of anti-apoptotic factor Bcl2 (Fig. 2C, G) was decreased. These alterations could be reversed by GEN treatment. Thus, GEN exhibited protective effects against CHO-induced osteoblast apoptosis.

### GEN inhibited CHO-induced ER stress and apoptosis in osteoblasts

**Fig. 3:**
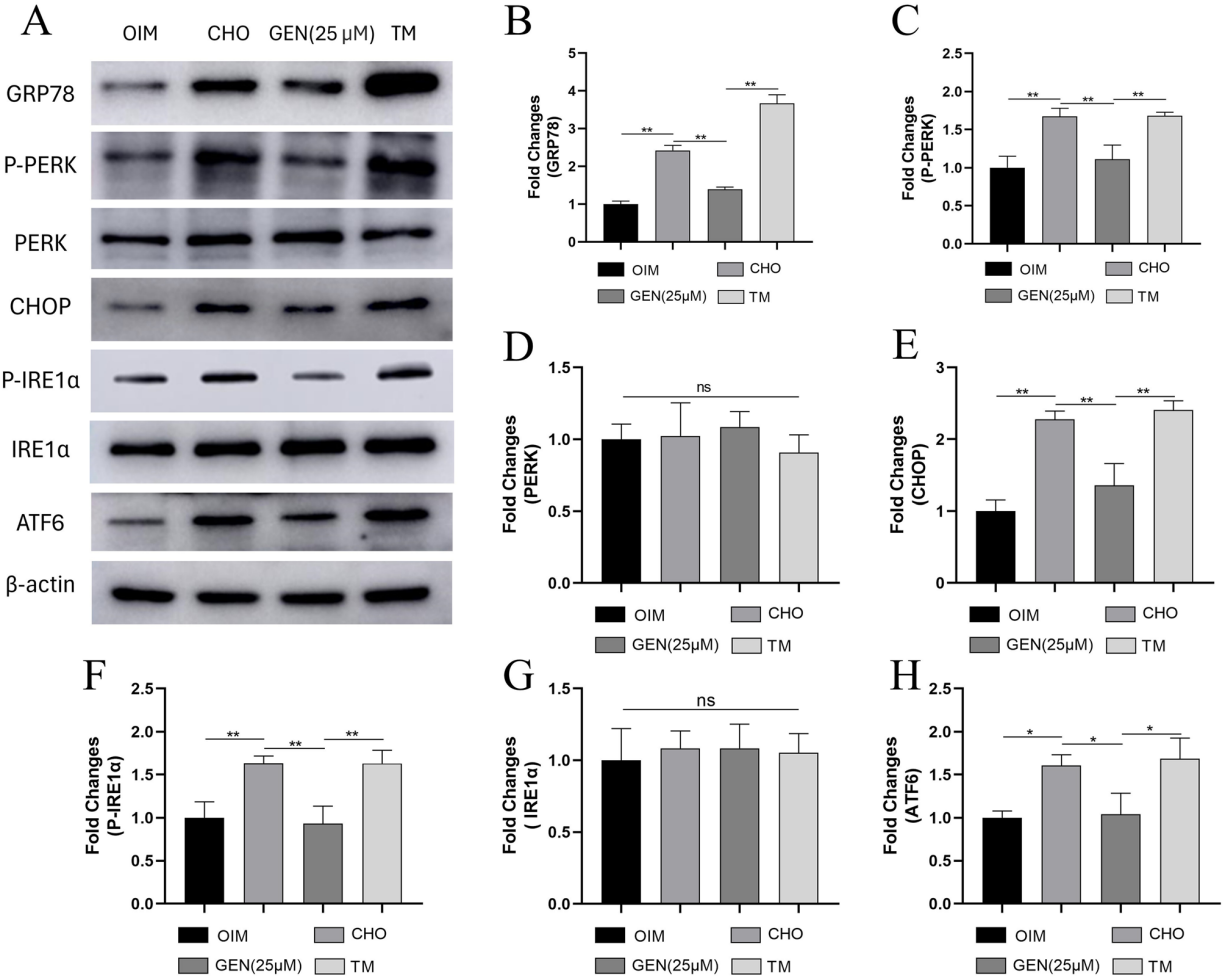
GEN inhibited CHO-induced ER stress in osteoblasts. The protein expressions of GRP78 (**A**,**B**), p-PERK (**A**,**C**), PREK (**A**,**D**), CHOP (**A**,**E**), p-IRE1α (**A**,**F**), IRE1α (**A**,**G**) and ATF6 (**A**,**H**) were analyzed by Western blot. * *p* < 0.05; ** *p* < 0.01; ns, no statistical difference. GEN (25 µM), CHO + GEN (25 µM); TM, CHO + GEN (25 µM) + TM (1.5 µg/ml)

**Fig. 4:**
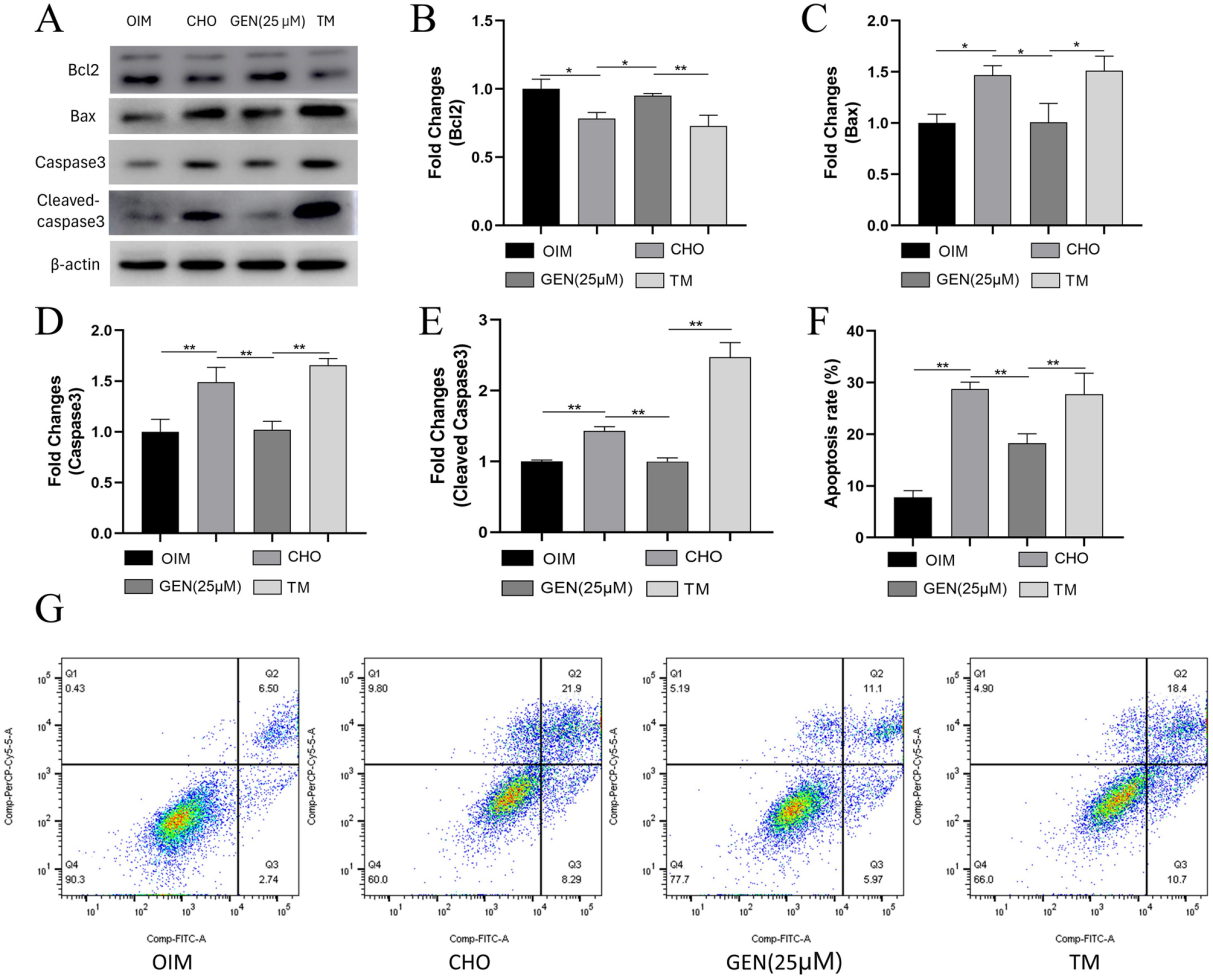
GEN inhibited CHO-induced apoptosis in osteoblasts by suppressing ER stress. The protein expressions of Bcl2 (**A**,**B**), Bax (**A**,**C**), caspase3 (**A**,**D**), and cleaved caspase3 (**A**,**E**) were analyzed by Western blot. (**F**,**G**) Apoptosis analysis was performed by flow cytometry. * *p* < 0.05; ** *p* < 0.01; ns, no statistical difference. GEN (25 µM), CHO + GEN (25 µM); TM, CHO + GEN (25 µM) + TM (1.5 µg/ml)

To further explore the potential mechanisms of GEN in protecting osteoblasts from CHO-induced damage, we examined the expression of ER stress-related proteins. The results showed that the expression levels of ER stress-related proteins GRP78 (Fig. 3A,B), p-PERK (Fig. 3A,C), CHOP (Fig. 3A,E), p-IRE1α (Fig. 3A,F), and ATF6 (Fig. 3A,H) were significantly increased in CHO-treated groups. However, the expression of PERK (Fig. 3A,D) and IRE1α (Fig. 3A,G) was not affected significantly. In addition, the expression levels of Bax (Fig. 4A,C), caspase3 (Fig. 4A,D), and cleaved caspase3 (Fig. 4A,E) were increased, and the expression of Bcl-2(Fig. 4A,B) was decreased. GEN attenuated ER stress in osteoblasts, as shown by decreased expression of GRP78, p-PERK, CHOP, p-IRE1α, and ATF6. Tunicamycin (TM) has been used as an agonist of ER stress. To explore whether GEN inhibited CHO-induced apoptosis by activating ER stress, TM was added for 24-h stimulation. TM was shown to significantly activate ER stress. TM could compromise the effects of GEN and reactivate ER stress to promote osteoblast apoptosis (Fig. 4F,G). Thus, GEN inhibited CHO-induced osteoblast apoptosis by suppressing ER stress.

### GEN inhibited CHO-induced ER stress and apoptosis by activating ABCA1 expression

**Fig. 5:**
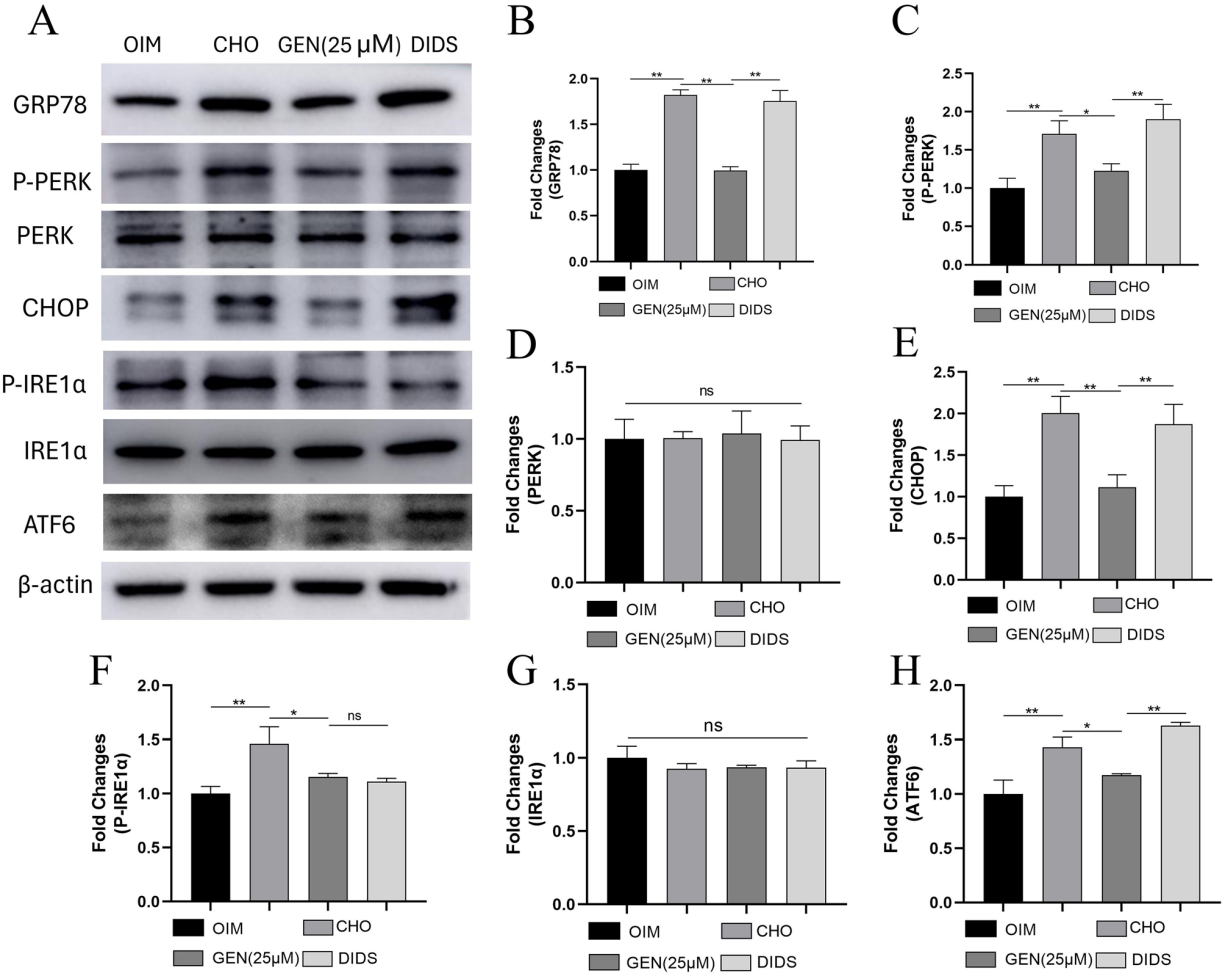
GEN inhibited CHO-induced ER stress in osteoblasts by activating ABCA1 expression. The protein expressions of GRP78 (**A**,**B**), p-PERK (**A**,**C**), PREK (**A**,**D**), CHOP (**A**,**E**), p-IRE1α (**A**,**F**), IRE1α (**A**,**G**) and ATF6 (**A**,**H**) were analyzed by Western blot. * *p* < 0.05; ** *p* < 0.01; ns, no statistical difference. GEN (25 µM), CHO + GEN (25 µM); DIDS, CHO + GEN (25 µM) + DIDS(20µM)

**Fig. 6:**
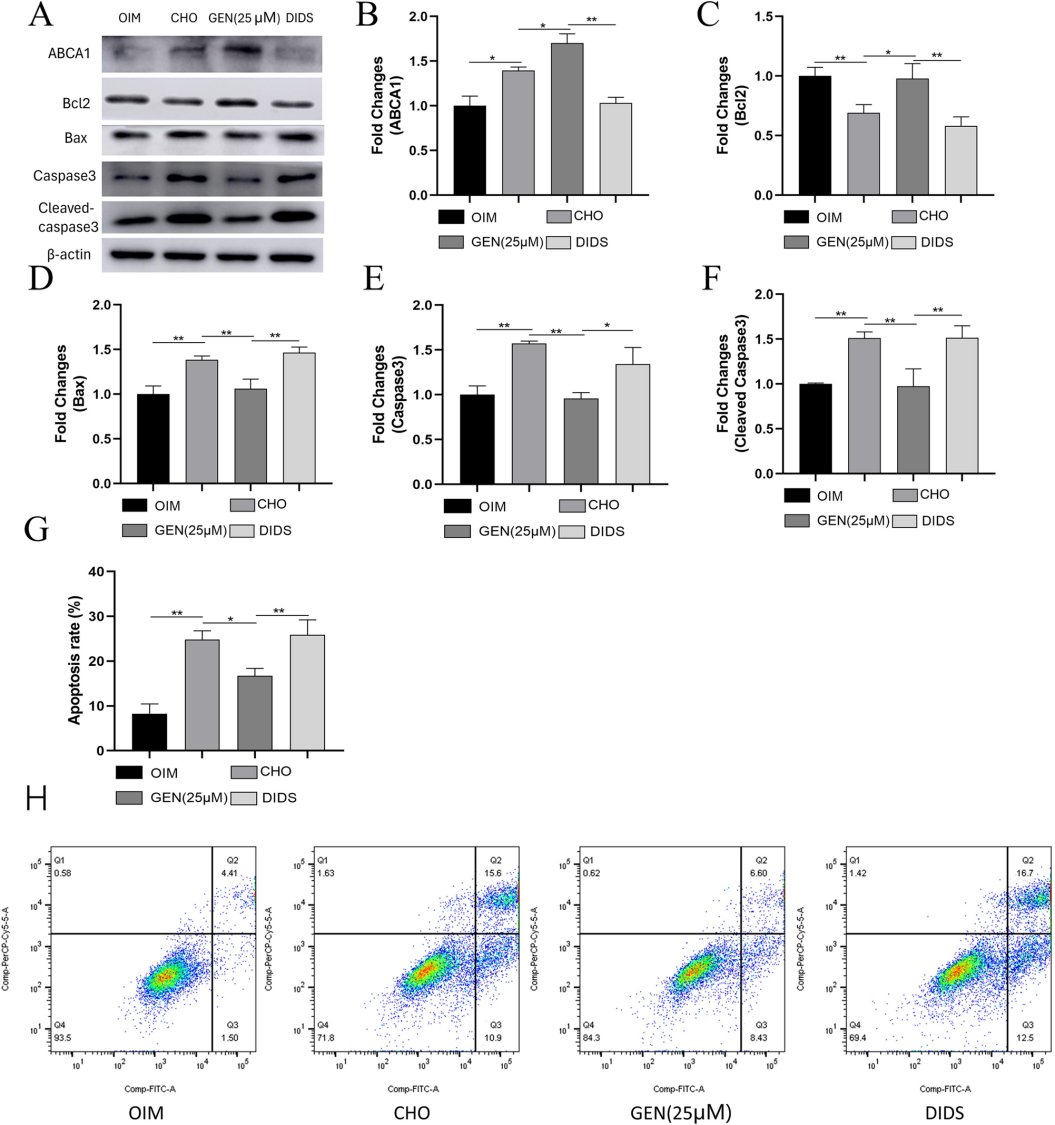
GEN inhibited CHO-induced apoptosis in osteoblasts by activating ABCA1 expression. The protein expressions of ABCA1 (**A**,**B**), Bcl2 (**A**,**C**), Bax (**A**,**D**), caspase3 (**A**,**E**), and cleaved caspase3 (**A**,**F**) were analyzed by Western blot. (**G**,**H**) Apoptosis analysis was performed by flow cytometry. * *p* < 0.05; ** *p* < 0.01; ns, no statistical difference. GEN (25 µM), CHO + GEN (25 µM); DIDS, CHO + GEN (25 µM) + DIDS (20µM)

Our previous study demonstrated that GEN promotes ABCA1 expression to reduce intracellular CHO accumulation. DIDS, an inhibitor of ABCA1, has been reported to suppress the activity of ABCA1, which may promote cholesterol efflux [25]. To further investigate how GEN affects CHO-induced ER stress in osteoblasts, the ABCA1 inhibitor DIDS was added for 72-h treatment. The results showed that DIDS could up-regulate the expression of GRP78 (Fig. 5A,B), p-PERK (Fig. 5A,C), CHOP (Fig. 5A,E), and ATF6 (Fig. 5A,H), neutralizing the effects of GEN. However, DIDS exhibited no effects on GEN-induced down-regulation of p-IRE1α (Fig. 5A,F). DIDS also did not affect the protein expression of PERK (Fig. 5A,D) and IRE1α (Fig. 5A,G). GEN increased the expression of ABCA1. However, DIDS eliminated GEN-mediated ABCA1 expression (Fig. 6A,B). In addition, DIDS abrogated the inhibitory activity of GEN on CHO-induced osteoblast apoptosis, as shown by decreased expression of Bcl2 (Fig. 6A,C), increased expression of Bax (Fig. 6A,D), caspase3 (Fig. 6A,E), and cleaved caspase3 (Fig. 6A,F), and enhanced apoptosis detected by flow cytometry (Fig. 6G,H). Thus, CHO accumulation could induce ER stress and osteoblast apoptosis, and GEN inhibited CHO-induced ER stress and apoptosis by activating ABCA1 expression.

### GEN inhibited CHO-induced ER stress and apoptosis by activating GLP-1R expression

**Fig. 7:**
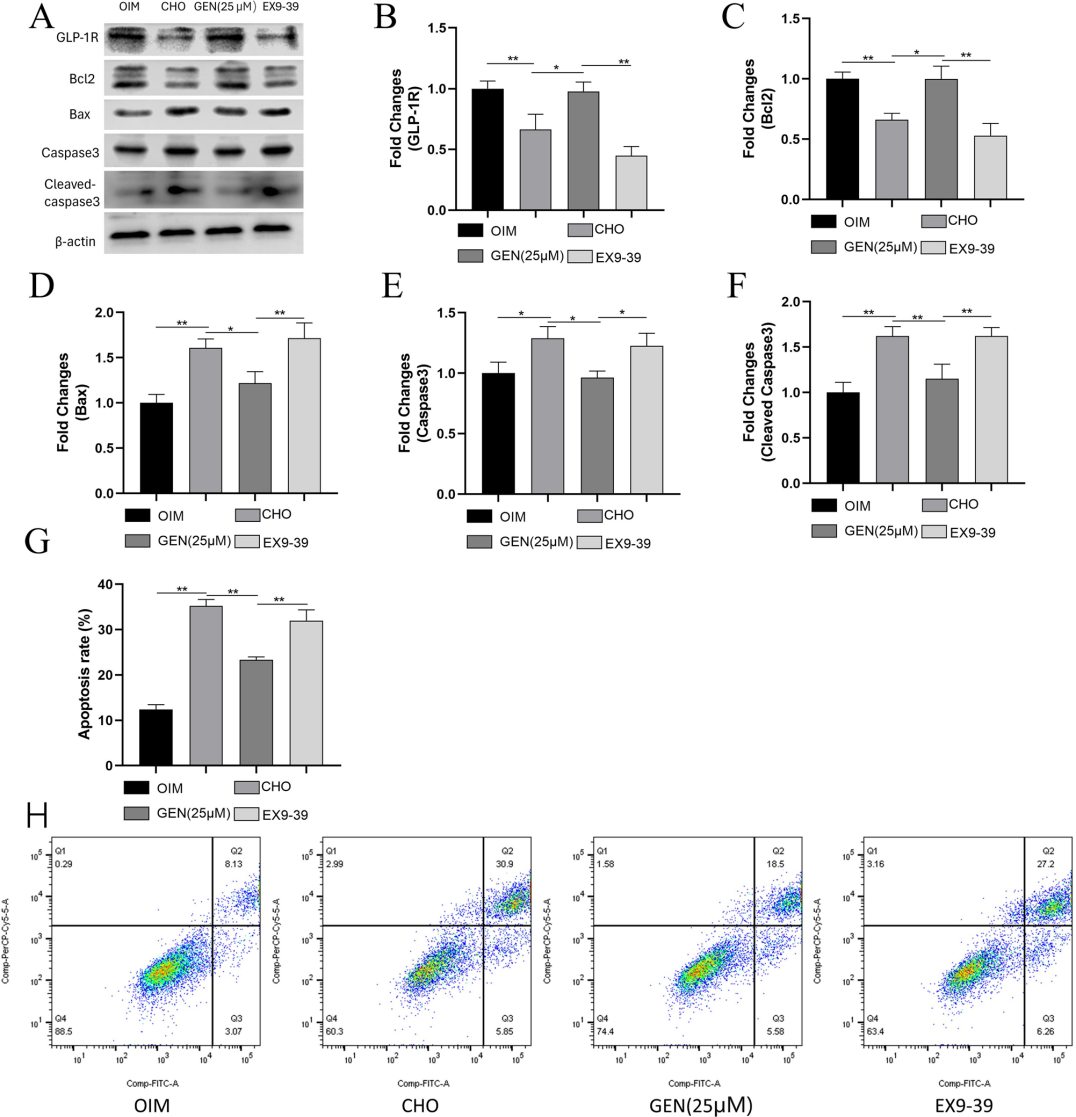
GEN inhibited CHO-induced apoptosis in osteoblasts by activating GLP-1R expression. The protein expressions of GLP-1R(**A**,**B**), Bcl2 (**A**,**C**), Bax (**A**,**D**), caspase3 (**A**,**E**), and cleaved caspase3 (**A**,**F**) were analyzed by Western blot. (**G**,**H**) Apoptosis analysis was applied by flow cytometry. * *p* < 0.05; ** *p* < 0.01; ns, no statistical difference. GEN (25 µM), CHO + GEN (25 µM); EX9-39, CHO + GEN (25 µM) + Exendin9-39 (200nM)

**Fig. 8:**
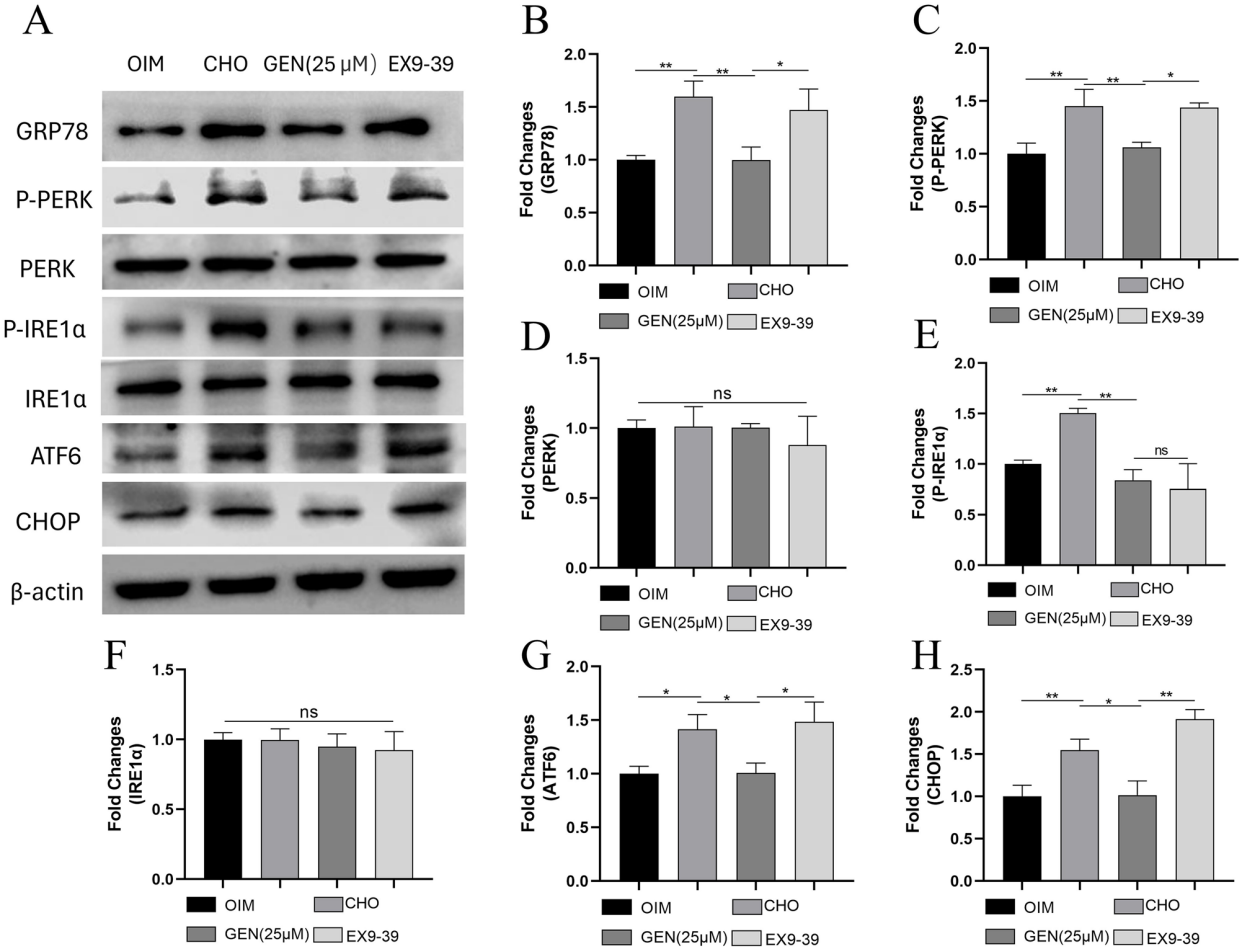
GEN inhibited CHO-induced ER stress in osteoblasts by activating GLP-1R expression. The protein expressions of GRP78 (**A**,**B**), p-PERK (**A**,**C**), PREK (**A**,**D**), p-IRE1α (**A**,**E**), IRE1α (**A**,**F**), ATF6(A,G), and CHOP(**A**,**H**) were analyzed by Western blot. * *p* < 0.05; ** *p* < 0.01; ns, no statistical difference. GEN (25 µM), CHO + GEN (25 µM); EX9-39, CHO + GEN (25 µM) + Exendin9-39 (200nM)

GEN can be an activator of GLP-1R [26], which has been reported to activate the downstream factor ABCA1 [27]. Exendin9-39 is the specific inhibitor of GLP-1R. Treatment with Exendin9-39 may abolish the biological effects of GLP-1R [28]. To further explore whether GLP-1R was implicated in the regulatory role of GEN in CHO-induced ER stress and osteoblast apoptosis, the GLP-1R inhibitor Exendin9-39 was added. The results showed that CHO down-regulated the expression of GLP-1R (Fig. 7A,B) in osteoblasts, which could be reversed by treatment with GEN. However, Exendin9-39 could inhibit the regulatory effects of GEN on GLP-1R expression. Exendin9-39 also eliminated the inhibitory activity of GEN on osteoblast apoptosis, as shown by decreased expression of Bcl2 (Fig. 7A,C) and increased expression of Bax (Fig. 7A,D), caspase3 (Fig. 7A,E), and cleaved-caspase3 (Fig. 7A,F). The effects of Exendin9-39 on osteoblast apoptosis (Fig. 7G,H) could be verified by flow cytometry. In addition, Exendin9-39 could activate ER stress, as indicated by up-regulated expression of GRP78 (Fig. 8A,B), p-PERK (Fig. 8A,C), p-IRE1α (Fig. 8A,E), ATF6 (Fig. 8A,G), and CHOP (Fig. 8A,H). Exendin9-39 did not affect the expression of PERK (Fig. 8A,D) and IRE1α (Fig. 8A,F) significantly. Thus, GEN inhibited CHO-induced ER stress and apoptosis by activating GLP-1R expression.

## Discussion

Previous studies have shown that high CHO levels in the body increase the risk of osteoporosis [7, 29–31]. High concentration of CHO is detrimental to osteoblast survival [32, 33]. However, the mechanism by which it induces osteoporosis has not been fully elucidated. Our previous studies indicate that high concentration of CHO inhibits the proliferation and differentiation of osteoblast and promote osteoporosis development [24, 34]. In the current study, we further found that high concentration of CHO induced ER stress and cell apoptosis in osteoblasts. GEN exhibited protective activity against ER stress and osteoblast apoptosis.

CHO is a nonpolar hydrophobic molecule that is essential for the performance of many cellular functions [35, 36]. CHO plays an essential role in bone metabolism [37]. For example, CHO supports cellular function. However, high CHO levels are detrimental to the survival of osteoblasts [38]. It has been shown that high CHO levels suppress the proliferation and differentiation of osteoblasts, inhibit bone formation, and promote bone resorption in a time- and dose-dependent manner [29]. CHO and its metabolites affect bone homeostasis by regulating the differentiation and activation of osteoblasts and osteoclasts [7, 39]. Therefore, lowering the level of cholesterol in osteoblasts can alleviate cholesterol-induced osteoporosis [37]. Probucol, a cholesterol-lowering drug, can promote osteogenic differentiation and delay the onset of osteoporosis [40]. GEN has been shown to inhibit CHO accumulation in osteoblasts. However, the mechanism is unclear.

The pathological development of osteoporosis can be promoted by ER stress and oxidative stress [14]. Oxysterols are cholesterol derivatives that have been shown to induce ER stress in different cell types and pathological contexts [41]. In addition, the ER is the site of synthesis of CHO and other important lipids, and cells undergoing CHO metabolic overload will develop ER stress [42]. Moreover, ER stress will further impair CHO metabolism, forming a vicious circle [8]. In our study, we found that CHO at the concentration of 100 µM could activate ER stress in osteoblasts, as indicated by up-regulating the expression of ER stress biomarkers GRP87 and CHOP. Inhibition of CHO accumulation can be the therapeutic strategy for osteoporosis treatment. Promoting CHO efflux can protect osteoblasts from pathological changes and promote osteoblast survival. GEN has been shown to reduce serum CHO levels and intracellular CHO accumulation [23]. Our study further demonstrated that GEN increased CHO efflux, inhibited ER stress, and reduced cell apoptosis in osteoblasts.

ABCA1 is a plasma HDL cholesterol regulator that primarily regulates CHO efflux [43]. GLP-1R is a G protein-coupled receptor that mediates intracellular signaling [44]. GLP-1R agonists are a class of drugs that are effective in treating or preventing obesity [45]. The interconnection between GLP-1R and ABCA1 has long been recognized, and GLP-1R is an upstream regulatory factor of ABCA1. It has been found that GLP-1R may positively regulate ABCA1 expression [46, 47]. ABCA1 regulates CHO levels and maintains intracellular CHO homeostasis [48, 49]. In this study, we found that there was a minor increase in the expression of ABCA1 in CHO-treated osteoblasts. This might be related to the self-protection mechanism of cells or some undiscovered mechanisms. In addition, GLP-1R expression was inhibited in CHO-treated osteoblasts.

GEN is a natural compound that has been found to have therapeutic benefits and treat a variety of diseases, including osteoporosis [50, 51]. GEN can up-regulate the expression of GLP-1R and ABCA1 in CHO-treated osteoblasts and promote CHO efflux from osteoblasts, suggesting that GEN might be the potential candidate for managing high-CHO-induced osteoporosis. Mechanically, the expression of ER stress-related proteins, such as GRP78 and CHOP, was down-regulated as the accumulation of CHO in osteoblasts decreased. DIDS, an inhibitor of ABCA1, inhibited the expression of ABCA1, increased in CHO accumulation in osteoblasts, and activate ER stress. Exendin (9–39), an antagonist of GLP-1R, neutralized the protective effects of GEN, which could activate GLP-1R. These suggested that the GLP-1R/ABCA1 axis can be an important target of GEN in protecting osteoblasts from CHO accumulation.

There are some limitations to this paper. There are certain levels of cholesterol in lipoproteins in FBS, and it might exhibit a weaker effect on cells. We did not exclude it, which might interrupt our analysis. It has been reported that rat serum cholesterol is about 1.87 mM [29], which is higher than the dose for our in vitro study. However, the in vivo study is much complex, and the biological system in vivo is quite different from that in vitro. Overall, cholesterol in the serum has become an obviously limited factor in the current study. GEN has been shown to activate autophagy and inhibit ER stress and oxidative stress [24, 51]. The mechanisms of CHO-induced apoptosis in osteoblasts are complex, including oxidative stress [52]. We only investigated the protective effect of GEN against CHO-induced apoptosis by inhibiting ER stress. The expression level of ABCA1 was significantly correlated with the total intracellular cholesterol concentration. However, we did not investigate how GEN modulated CHO efflux from osteoblasts via ABCA1. In addition, we also did not further study the effects of GEN on osteoblast differentiation, proliferation, and mineralization after CHO treatment. This can be our next scientific plan in the near future. More studies are still needed.

## Conclusion

In this study, we found that CHO could induce osteoblast apoptosis and activate ER stress. GEN exhibited protective effects against CHO-induced ER stress and apoptosis in osteoblasts. Inhibition of ABCA1 or GLP-1R could neutralize the protective activity of GEN, promoting ER stress and osteoblast apoptosis. Therefore, GEN alleviated CHO-induced ER stress and osteoblast apoptosis by activating the GLP-1R/ABCA1 signaling pathway.

## Data Availability

The data used to support the findings of this study are included within the article.
